# Conservation planning for freshwater–marine carryover effects on Chinook salmon survival

**DOI:** 10.1002/ece3.3663

**Published:** 2017-11-28

**Authors:** Jennifer L. Gosselin, Richard W. Zabel, James J. Anderson, James R. Faulkner, António M. Baptista, Benjamin P. Sandford

**Affiliations:** ^1^ School of Aquatic and Fishery Sciences University of Washington Seattle WA USA; ^2^ Northwest Fisheries Science Center National Marine Fisheries Service National Oceanic and Atmospheric Administration Seattle WA USA; ^3^ Oregon Health and Science University Portland OR USA; ^4^ Northwest Fisheries Science Center National Marine Fisheries Service National Oceanic and Atmospheric Administration Pasco WA USA

**Keywords:** cross‐life stage and cumulative effects, delayed mortality, hydropower dams, real‐time monitoring, translocation

## Abstract

Experiences of migratory species in one habitat may affect their survival in the next habitat, in what is known as carryover effects. These effects are especially relevant for understanding how freshwater experience affects survival in anadromous fishes. Here, we study the carryover effects of juvenile salmon passage through a hydropower system (Snake and Columbia rivers, northwestern United States). To reduce the direct effect of hydrosystem passage on juveniles, some fishes are transported through the hydrosystem in barges, while the others are allowed to migrate in‐river. Although hydrosystem survival of transported fishes is greater than that of their run‐of‐river counterparts, their relative juvenile‐to‐adult survival (hereafter survival) can be less. We tested for carryover effects using generalized linear mixed effects models of survival with over 1 million tagged Chinook salmon, *Oncorhynchus tshawytscha* (Walbaum) (Salmonidae), migrating in 1999–2013. Carryover effects were identified with rear‐type (wild vs. hatchery), passage‐type (run‐of‐river vs. transported), and freshwater and marine covariates. Importantly, the Pacific Decadal Oscillation (PDO) index characterizing cool/warm (i.e., productive/nonproductive) ocean phases had a strong influence on the relative survival of rear‐ and passage‐types. Specifically, transportation benefited wild Chinook salmon more in cool PDO years, while hatchery counterparts benefited more in warm PDO years. Transportation was detrimental for wild Chinook salmon migrating early in the season, but beneficial for later season migrants. Hatchery counterparts benefited from transportation throughout the season. Altogether, wild fish could benefit from transportation approximately 2 weeks earlier during cool PDO years, with still a benefit to hatchery counterparts. Furthermore, we found some support for hypotheses related to higher survival with increased river flow, high predation in the estuary and plume areas, and faster migration and development‐related increased survival with temperature. Thus, pre‐ and within‐season information on local‐ and broad‐scale conditions across habitats can be useful for planning and implementing real‐time conservation programs.

## 1 INTRODUCTION

Carryover effects are often overlooked in management and conservation of migratory species (O'Connor & Cooke, 2015; O'Connor, Norris, Crossin, & Cooke, 2014). These effects are how experiences in one habitat change performance in the next habitat. For example, the amount and quality of food available affect an individual's fat reserves and can in turn affect its later survival and reproductive success. Thus, conservation can be improved if the effect of one habitat on the next is considered. These indirect effects have been documented across wide‐ranging taxa including birds (Duriez, Ens, Choquet, Pradel, & Klaassen, 2012; Studds & Marra, 2005), mammals (Davy et al., 2016), amphibians (Chelgren, Rosenberg, Heppell, & Gitelman, 2006), reptiles (Ceriani et al., 2015), invertebrates (Hettinger et al., 2012), and fishes (Brosnan, Welch, Rechisky, & Porter, 2014; Russell et al., 2012).

To date, most carryover effects documented have been related to early‐life effects on reproductive success (Harrison, Blount, Inger, Norris, & Bearhop, 2011). Survival effects have received less attention because of the challenges in tracking individuals and recording conditions across habitats. Recent technological advances have diminished some of these challenges with increased accessibility to environmental data, usage of biologging, and availability of sufficiently long time series (Bograd, Block, Costa, & Godley, 2010; Drenner et al., 2012). It is now possible for preseason and within‐season management decisions to include these types of information more effectively. In this study, we take advantage of an extensive dataset of tagged fish to examine carryover effects. Nonetheless, the general patterns and concept of carryover effects can be applicable to other migratory species.

**Figure 1:**
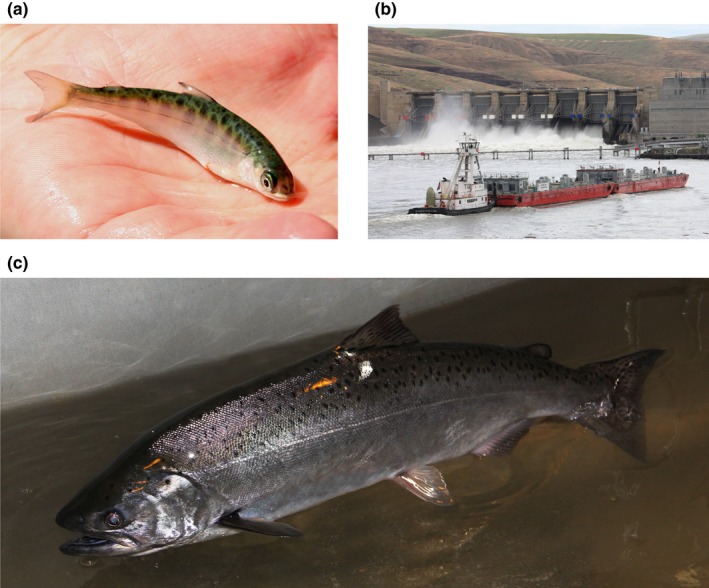
Chinook salmon (a) tagged as a juvenile, (b) transported in a barge at Lower Granite Dam, and (c) returned as an adult to Lower Granite Dam. Photograph credit: Benjamin P. Sandford

Conservation of anadromous fish species is complex because, by definition, their life cycle spans both freshwater and marine environments. While the marine environment exerts broad and strong effects on survival (Mantua, Hare, Zhang, Wallace, & Francis, 1997; Rupp, Wainwright, Lawson, & Peterson, 2012), carryover effects from the river environment are also important (Russell et al., 2012). In the highly human‐modified river system of the Snake and Columbia rivers (Idaho, Washington and Oregon, USA), several evolutionary significant units of salmon and steelhead, *Oncorhynchus* species (Walbaum) (Salmonidae), are listed under the U.S. Endangered Species Act (NMFS 2010). Hundreds of millions of U.S. dollars are spent annually in conservation efforts to reduce direct mortality during passage through multiple hydropower dams. The Juvenile Fish Transportation Program (USACE 2016) is one of the major conservation efforts designed to mitigate the effects of dam passage (Figure 1). However, the program has mixed success (Dietrich et al., 2016; Holsman, Scheuerell, Buhle, & Emmett, 2012). The direct survival of Chinook salmon through the hydropower system can be increased from 40–60% (DeHart et al., 2015; Faulkner, Widener, Smith, Marsh, & Zabel, 2016) to nearly 100% (McMichael, Skalski, & Deters, 2011). However, transported fish can suffer higher rates of posthydrosystem mortality than their run‐of‐river counterparts (DeHart et al., 2015; Smith, Marsh, Emmett, Muir, & Zabel, 2013). This can result in beneficial and detrimental net effects on adult salmon returns (reviewed in Anderson, Ham, & Gosselin, 2012). The juvenile‐to‐adult survival of transported fish also depends on rear‐types, with generally greater advantages to hatchery fish relative to wild fish. In essence, variation in survival can be attributed to three major factors: rear‐type (wild vs. hatchery), passage‐type (run‐of‐river vs. transported), and conditions experienced.

Linking these factors of salmonid survival involves riverine, estuarine, coastal, and oceanic conditions (Brosnan et al., 2014; Holsman et al., 2012; Miller, Teel, Peterson, & Baptista, 2014; Scheuerell, Zabel, & Sandford, 2009). At the crux of carryover processes is ocean arrival timing of juveniles (or smolts) as they transition from freshwater to marine environments. The timing of the transition is an important predictor of Snake River Chinook salmon survival (Petrosky & Schaller, 2010; Scheuerell et al., 2009). This migration timing is dependent on river temperatures and flows the juveniles experienced. Additionally, temperature affects growth, metabolic rates, development, behavior, and predator–prey interactions (McCullough et al., 2009). Thus, transportation that reduces juvenile river passage from weeks to days can strongly affect their timing of ocean entry, fish condition, and survival.

While mortality is high in the coastal ocean (Brosnan et al., 2014), the greater prey resources also afford higher growth than in the river (Burke et al., 2013; Weitkamp et al., 2015). Growth and survival have been related to indices of local marine conditions, such as an upwelling index (Logerwell, Mantua, Lawson, Francis, & Agostini, 2003; Scheuerell & Williams, 2005) and sea surface temperatures (Drenner et al., 2012; Miller et al., 2014). These processes involve shifts in the abundance and quality of the copepod and ichthyoplankton forage base (Daly, Auth, Brodeur, & Peterson, 2013; Peterson et al., 2014). These occur through variations in the horizontal advection of oceanic surface water to the near habitat, and upwelling within the habitat; both of which vary with the PDO index (Bi, Peterson, & Strub, 2011). Beyond the local environment, broad‐scale variations of the PDO (Mantua et al., 1997), North Pacific Gyre Oscillation (NPGO) (Di Lorenzo et al., 2008), and Multivariate El Niño‐Southern Oscillation (Wolter & Timlin, 1993) indices correlate to measures of adult salmon abundances, survival, productivity, and growth (Burke et al., 2013; Peterson et al., 2014; Rupp et al., 2012; Wells, Grimes, Field, & Reiss, 2006). Thus, understanding and predicting salmonid survival in the early ocean environment require information at local and basin scales, and at seasonal and annual scales (Brosnan et al., 2014; Duffy & Beauchamp, 2011; Weitkamp et al., 2015; Wells et al., 2016).

The goal of this study was to investigate the carryover effects of salmon experience during hydrosystem passage on their juvenile‐to‐adult survival. This study demonstrates that the largest influences on survival involve seasonal river migration timing and the phase of the PDO index. The local freshwater and marine covariates have weaker effects on survival and differ among rear‐types and passage‐types. Most notably, the analysis demonstrates that benefits of the juvenile transportation for wild and hatchery salmon differ and depend on the phase of the PDO index.

## 2 MATERIALS AND METHODS

We examined Chinook salmon survival with generalized linear mixed effects models (Zuur, Ieno, Walker, Saveliev, & Smith, 2009). We grouped covariates in a cumulative manner that reflected their life cycle from the freshwater to the marine environment: the migration–timing models (MT) incorporated day of year (DOY) of passage at Bonneville Dam (BON) as an index; the freshwater models (MT–FW) added local, seasonal river conditions; the marine models (MT–FW–M) added local, estuarine, plume, and coastal ocean conditions; and the climate models (MT–FW–M–C) added a categorical index of large‐scale, climate‐influenced, marine conditions. Notably, the local covariates tested are collected in real time, and the large‐scale, climate covariate can be predicted at a coarse scale several months in the future (Newman et al., 2016). Therefore, the covariates tested can be used in real time for management of juveniles migrating through the hydropower system, and the marine and climate conditions are not considered without freshwater conditions. Also, our analysis builds on the results from Holsman et al. (2012), Scheuerell et al. (2009), and Satterthwaite et al. (2014) that found migration timing to be an important predictor of survival.

### 2.1 Data

#### 2.1.1 Fish samples and treatment groups

We analyzed spring/summer runs of Chinook salmon from the Snake River system. These individuals originated above Lower Granite Dam (LGR) and migrated to the ocean in years 1999–2013. We tested different treatment groups by combinations of rear‐type (wild or hatchery) and passage‐type (run‐of‐river or transported). For each treatment group, we modeled its survival from its own dataset. The fish were tagged with passive‐integrated transponder (PIT) tags, and these data are publically available through the Columbia Basin PIT Tag Information System (www.ptagis.org). All run‐of‐river fish in the dataset were detected at BON, the last dam encountered during their outmigration. Transported fish were loaded onto barges at LGR and then transported to a release site downstream of BON. Survival was calculated from juvenile passage at BON or release below BON to adult returns at LGR. LGR was chosen as the adult detection site to account for any increased probability of straying during upstream migration in transported fishes (Bond et al., 2017; Keefer & Caudill, 2014). Thus, juvenile‐to‐adult survival in this study includes successful return of adults to LGR. Furthermore, only juveniles passing BON between DOY 100 and 180 were included in the analysis because of insufficient numbers of fish early and late in the season. See Figures S1 and S2, for yearly sample sizes of juveniles and adults.

#### 2.1.2 Fish and environmental covariates

The fish and freshwater covariates from the first habitat were tested for carryover effects, while marine and climate covariates from the second habitat were tested for direct effects and their moderation of first habitat carryover effects (Table 1).

**Table 1:** Model covariates related to each juvenile by day of passage at BON were grouped as migration timing (MT), freshwater (F), marine (M), or climate (C) covariates

Name	Symbol	Description	Units	Source of data	Covariate group
Migration–timing index	*d*	DOY when passage at BON occurred	day	www.ptagis.org/	MT
River temperature	*t*	Residual effect of river temperature WQM at BON, after controlling for *d*	°C	http://www.cbr.washington.edu/dart/river.html	F
River flow	*f*	Flow at BON when passage occurred	kcfs	http://www.cbr.washington.edu/dart/river.html	F
Sea surface temperature	*T*	Residual effect of 7‐day rolling mean of sea surface temperature from NDBC buoys (stations lapw1, 46211, 46041, 46029, and 46050), after controlling for *d*	°C	www.ndbc.noaa.gov or http://www.cbr.washington.edu/dart/buoy_com.html	M
Coastal upwelling index	*U*	7‐day rolling mean of coastal upwelling index at 45°N 125°W	m^3^ per second per 100 m of coastline	http://www.pfeg.noaa.gov/ or http://www.cbr.washington.edu/dart/upwell_com.html	M
Estuary salt intrusion length	*E*	Residual effect of 7‐day rolling mean of maximum along channel distance upstream of the river mouth where salinity ≥1 practical salinity unit, after accounting for flow *f*	km	http://www.stccmop.org/datamart/virtualcolumbiariver/simulationdatabases/climatologicalatlas_db33	M
Plume volume	*V*	Residual effect of 7‐day rolling mean of plume volume, after accounting for flow *f*	m^3^	http://www.stccmop.org/datamart/virtualcolumbiariver/simulationdatabases/climatologicalatlas_db33	M
Categorical PDO index	*I*	1 for favorable ocean conditions with PDO < 0, and 0 for unfavorable ocean conditions with PDO > 0.	unitless	http://research.jisao.washington.edu/pdo/PDO.latest	C

##### Migration timing

The covariate DOY of BON passage was tested for linear (i.e., *d*) and nonlinear (i.e., *d* ^2^) patterns (PIT Tag Information System, ptagis.org).

##### Freshwater covariates

The freshwater covariates were river flow (*f*) and the residual effect of river temperature (*t*) measured on the day of passage at BON (U.S. Army Corps of Engineers, accessed via www.cbr.washington.edu/dart/river.html). Because of high correlation between river temperature and *d* (Table S1), a residual effect *t* was calculated as residuals from the linear regression between river temperature and *d*.

##### Marine covariates

The estuarine and marine environmental covariates were the coastal upwelling index (*U*) at 45°N 125°W (Pacific Fisheries Environmental Laboratory, www.pfeg.noaa.gov) and the residual effect of mean sea surface temperature (*T*) across five stations in the coastal ocean near Columbia River (National Data Buoy Center, www.ndbc.noaa.gov) after accounting for the migration timing index *d*. We also tested the salt intrusion length (*E*) as a covariate of the Columbia River estuary, and the volume (*V*) as a covariate of the Columbia River plume (Climatological Atlas for DB33, www.stccmop.org). Covariates *E* and *V* are products of the Virtual Columbia River (Baptista et al., 2015) that were computed from numerical simulations of 3D baroclinic circulation (Kärnä & Baptista, 2016). Because the migration timing through the estuary and coastal ocean was not observed, we used a 7‐day rolling mean right‐aligned to *d* for these marine covariates based on estimates reviewed in Dietrich et al. (2016).

##### Climate covariate

The PDO index exhibits oscillatory patterns that represent large‐scale marine and climate conditions (Mantua et al., 1997; Newman et al., 2016; Peterson et al., 2014). A negative PDO index represents relatively cool sea surface temperatures along the Pacific Coast. Conversely, a positive PDO index represents relatively warm coastal sea surface temperatures. The cool/warm phases of the PDO index can be predictive of the prey resources and predators (Emmett & Krutzikowsky, 2008; Peterson et al., 2014).

Although we do not know the value of the PDO index that the salmon will experience months into the future, we can at least reasonably predict whether the index will be positive or negative based on recent trends. Newman et al. (2016) showed an autocorrelation of at least 0.5, with a lag of up to 6 months. Such a binary index is reasonable given the strong and divergent effects from opposing climate phases and the presence of thresholds or tipping points (Hunsicker et al., 2016; Samhouri et al., 2017).

We determined a binary PDO index based on the mean PDO index May through September in the year of outmigration. In the model, the negative values of the mean PDO index was scored as *I* = 1 to represent cool and favorable conditions. Conversely, positive values of the mean PDO index was *I* = 0 to represent warm and unfavorable conditions.

### 2.2 Analysis

#### 2.2.1 Survival

The survival predicted from the models, expressed as a probability of a juvenile at BON returning as an adult to LGR (i.e., Bernoulli trial), was estimated with a generalized linear mixed effects model (GLMM; Zuur et al., 2009):

(1) $$y_{\mathrm{ij}}∼\text{Bernoulli}\left(p_{\mathrm{ij}}\right)$$

(2) $$\text{logit}\left(p_{\mathrm{ij}}\right)=\beta_{0}+b_{0j}+\left(\beta_{1}+b_{1j}\right)d_{i}+\left(\beta_{2}+b_{2j}\right)d_{i}^{2}+\sum_{k=3}^{K}\beta_{k}x_{\mathrm{ki}}$$

$$b_{0j}∼N\left(0,\sigma_{0}\right)$$

$$b_{1j}∼N\left(0,\sigma_{1}\right)$$

$$b_{2j}∼N\left(0,\sigma_{2}\right)$$

where we have the binary outcome *y*_*ij*_ of whether or not individual *i* (*i* = 1, …, *n*) returns as an adult with probability *p*_*ij*_ in a Bernoulli distribution, fixed effect intercept β_0_, random effect intercept *b*_0*j*_ for year *j*, fixed slope β_1_ and random slope *b*_1*j*_ in year *j* for covariate *d*, fixed slope β_2_ and random slope *b*_2*j*_ in year *j* for covariate *d* ^2^, and fixed slope β_*k*_ for covariate *k* (i.e., all covariates in Table 1, except for *d*), in which *x*_*k*_ is the measured value of covariate *k* for individual *i*. Random effects were assumed independent and normally distributed with zero means and constant respective variances σ. Fixed effect covariates, except *I*, were standardized to a mean of 0 and standard deviation 1 for improved model fitting and convergence.

All possible combinations of covariates in Equation (2) were tested, with the exception that models with a quadratic term (*d* ^2^) also included a linear term (*d*). We tested random intercept effects of year and random slope effects of year for the migration timing covariates (*d* and *d* ^2^) but not for other covariates. Model averaging was determined using the bias‐corrected Akaike information criterion for small sample sizes (AICc; Burnham & Anderson, 2002), and by the weighted average of the predictions $\overset{¯}{Y}$, conditional on the covariate being present in model *m*, $\overset{¯}{Y}=\sum_{m=1}^{M}\omega_{m}Y_{m}$ *,* where *M* is the total number of models (i.e., *M* = 768), and ω_*m*_ is the ∆AICc–based weight proportional to 1: $\omega_{m}=\frac{\exp\left(-\frac{\Delta {\mathrm{AICc}}_{m}}{2}\right)}{\sum_{m=1}^{M}\exp\left(-\frac{\Delta {\mathrm{AICc}}_{m}}{2}\right)}$. The associated weights were used to determine the 99% confidence set of models for each “rear‐type × passage‐type” treatment group. To assess the relative importance of models at each cumulative grouping of covariates, we determined the weights for models associated with each grouping while excluding models from lower‐level groupings (e.g., MT–FW–M grouping excluded models in MT and MT–FW groupings). The model‐averaged parameters and relative importance of covariates across models in the 99% confidence set were determined. The analysis was conducted in R© 2016 The R Foundation for Statistical Computing (version 3.3.2) with the glmer function from the lme4 package (version 1.1‐12) and the model.avg function from the MuMIn package (version 1.15.6).

#### 2.2.2 Effectiveness of transportation program

A ratio of survival (*S*) for transported to run‐of‐river fish (i.e., differential delayed mortality, *D = S*_transport_ */S*_run‐of‐river_), characterizes the effectiveness of the juvenile fish transportation program after fish have passed BON (reviewed in Anderson et al., 2012). Thus, *D *>* *1 indicates an advantage from transportation on posthydrosystem survival, while *D *=* *1 indicates no effect, and *D *<* *1 indicates a detrimental effect. To determine the effect of transportation from LGR on survival, relative to that of run‐of‐river counterparts, we would need to incorporate the hydropower system survival of transported fish approximating 100% (McMichael et al., 2011) and the hydropower system survival of run‐of‐river fish approximating 50% (DeHart et al., 2015; Faulkner et al., 2016). *D* was thus compared to a threshold of 0.5 instead of 1 (reviewed in Anderson et al., 2012).

Patterns of *D* were determined from simulations based on our model‐averaged GLMMs of survival. We simulated parameters from the sampling distributions of the maximum likelihood estimates from our GLMMs, where the sampling distributions were assumed to be multivariate normal with means equal to the estimated regression parameters and covariances equal to the associated estimated covariance matrices for the parameters. These simulations were run in R© 2016 The R Foundation for Statistical Computing (version 3.3.2) with the sim function in the arm package (version 1.9‐3). Furthermore, to obtain model‐averaged survival estimates, a simulation was run for each candidate model of the confidence set, and the predicted survival probabilities were weighted by ω_*m*_ accordingly. To determine patterns of *D* with fixed effects only, we first generated a set of 1,000 model‐averaged simulations for each passage‐type and rear‐type combination based on draws of fixed effect parameters only. Then, we also included the random effects from the simulated sets estimated for the 15 years of data. Finally, to visualize patterns of *D* in cool and warm PDO years separately, we applied the simulated parameters to the average observed values of covariates across years with *I* = 1 or 0, respectively.

## 3 RESULTS

**Figure 2:**
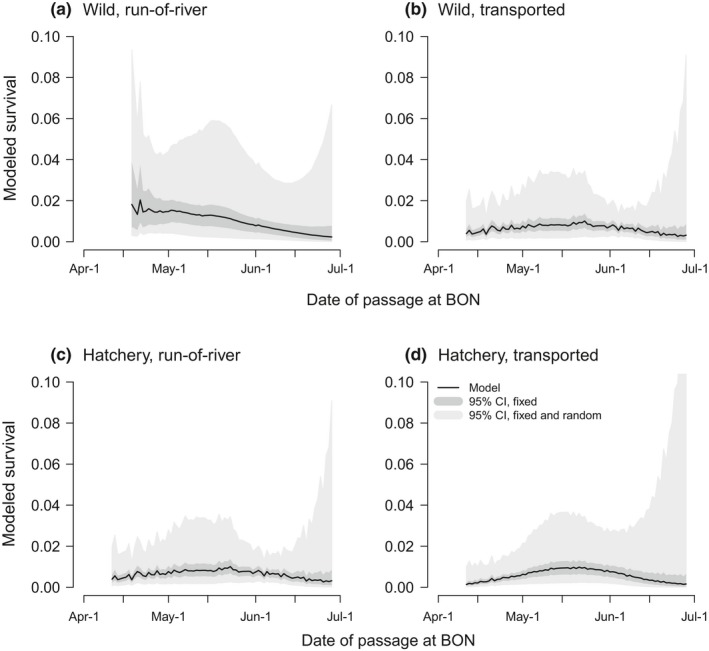
Modeled survival through outmigration seasons 1999–2013 for wild/hatchery, run‐of‐river/transported Chinook salmon. CI represents confidence interval. See Figure 3 for model parameters and relative importance of covariates

**Figure 3:**
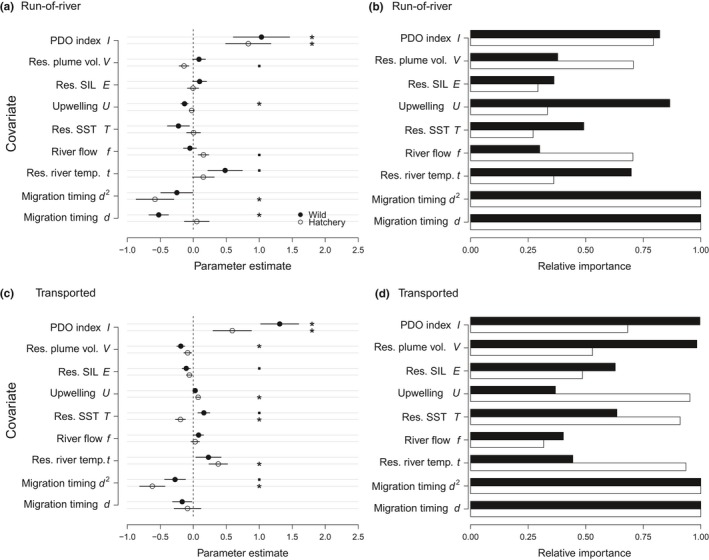
Standardized parameter estimates and relative importance of covariates in model‐averaged generalized linear mixed effects modeling of survival for run‐of‐river and transported Chinook salmon. Covariates are described in Table 1. Error bars represent standard deviation. Statistical significance denoted as * for *p* < .05 and • for *p* < .1

**Figure 4:**
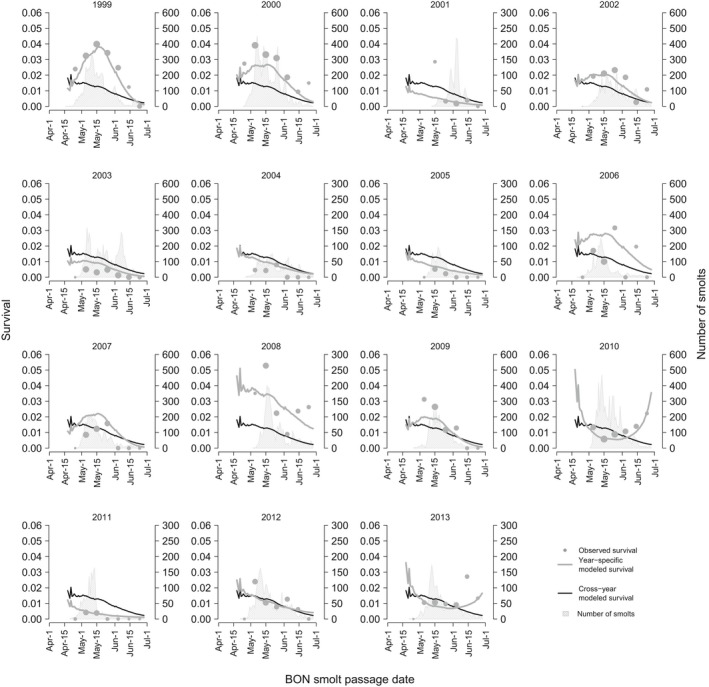
Wild, run‐of‐river Chinook salmon survival observed (passive‐integrated transponder‐tagged) and modeled (model‐averaged generalized linear mixed effects model, GLMM) estimates for each outmigration season 1999–2013. Gray points represent weekly observed estimates of survival. The size of points is representative of weekly juvenile sample sizes, as denoted numerically by light gray shading of daily smolt run. See Figures S3–S5 for other rear‐types and passage‐types of Chinook salmon

Across both rear‐types and both passage‐types, the PDO index *Ι* and migration timing indices *d* and/or *d* ^2^ were the most influential predictors (Figures 2 and 3; Table 2). Interannual differences in survival were thus explained by *Ι*, in which the cool phase implies favorable ocean conditions. The seasonal survival patterns were captured by *d* ^2^ for all rear‐type and passage‐type combinations, except wild run‐of‐river Chinook salmon. The model‐averaged fits thus generally showed a dome‐shaped pattern but also other nonlinear patterns (Figures 2 and 4; Figures S3–S5). Starting in mid‐April, survival generally increased until about mid‐May and decreased thereafter through June. In contrast, wild run‐of‐river Chinook survival declined through the season. Overall, *Ι* characterizes the interannual survival variation, and *d* and *d* ^2^ characterize seasonal survival variations.

**Table 2:** Number of models, minimum and maximum ∆AICc, and weight for the 99% confidence set, and all models tested in parentheses. Results reported are for models at each cumulative grouping of covariates (i.e., MT, MT–FW, MT–FW–M, and MT–FW–M–C), excluding models in lower‐level groupings. For each rear‐type and passage‐type combination, the cumulative grouping of covariates with greatest weight is bolded

Cumulative grouping of covariates	Number of models	Minimum ΔAICc	Maximum ΔAICc	Weight
(a) Wild, run‐of‐river Chinook				
MT	1 (6)	7.255 (7.255)	7.255 (65.529)	0.0022
MT‐FW	3 (18)	8.118 (8.118)	9.918 (65.226)	0.0028
MT‐FW‐M	56 (360)	2.632 (2.632)	12.260 (69.448)	0.1693
**MT‐FW‐M‐C**	**78 (384)**	**0.000 (0.000)**	**12.389 (67.506)**	**0.8158**
(b) Wild, transported Chinook				
MT	0 (6)	– (25.207)	– (97.816)	0.0000
MT‐FW	0 (18)	– (19.219)	– (98.612)	0.0000
MT‐FW‐M	0 (360)	– (11.365)	– (104.495)	0.0000
**MT‐FW‐M‐C**	**40 (384)**	**0.000 (0.000)**	**9.575 (100.776)**	**0.9903**
(c) Hatchery, run‐of‐river Chinook				
MT	1 (6)	10.850 (10.850)	10.850 (80.327)	0.0004
MT‐FW	3 (18)	3.987 (3.987)	10.387 (61.156)	0.0176
MT‐FW‐M	53 (360)	2.808 (2.808)	10.964 (81.404)	0.1831
**MT‐FW‐M‐C**	**75 (384)**	**0.000 (0.000)**	**11.068 (80.534)**	**0.7890**
(d) Hatchery, transported Chinook				
MT	0 (6)	– (20.079)	– (577.703)	0.000
MT‐FW	0 (18)	– (16.441)	– (577.987)	0.000
MT‐FW‐M	26 (360)	1.317 (1.317)	9.464 (581.162)	0.3085
**MT‐FW‐M‐C**	**40 (384)**	**0.000 (0.000)**	**9.2401 (578.800)**	**0.6816**

While the interannual and seasonal variations (Figures 4, S3–S5) were largely captured by *Ι* and *d*, local riverine and marine seasonal covariates also contributed to the variations (Figure 3). The wild run‐of‐river Chinook salmon survival (Figure 3a,b) was influenced positively by residual river temperature *t* and negatively by upwelling index *U*. Thus, wild fish appeared to benefit from both relatively warmer temperatures in the river and sea surface as weak upwelling can result in warmer coastal sea surface temperatures. Note that the temperature index is a residual after accounting for the negative effects from later migration timing and correlated warmer temperatures. The hatchery run‐of‐river survival was influenced positively by flow *f* and negatively by residual plume volume *V* after accounting for flow. These patterns suggest that wild Chinook salmon are responding to temperature‐related covariates, while the hatchery run‐of‐river fish are responding to flow‐related covariates.

For transported Chinook salmon (Figure 3c,d), wild fish survival was influenced by local marine covariates. In contrast, hatchery fish survival was influenced by temperature‐related covariates in both river and marine environments. Specifically, wild fish benefited from a positive residual sea surface temperature *T* after accounting for migration timing. The positive effect of *T* may involve faster migration rates and development. Also, the negative effect from the residual plume volume *V* suggests that the plume is an area of relatively high mortality. For the hatchery counterparts, survival improved with a positive residual river temperature *t*, a negative residual sea surface temperature *T*, and a positive upwelling index *U*. Together, these patterns show differing effects on survival across rear‐types and passage‐types.

**Figure 5:**
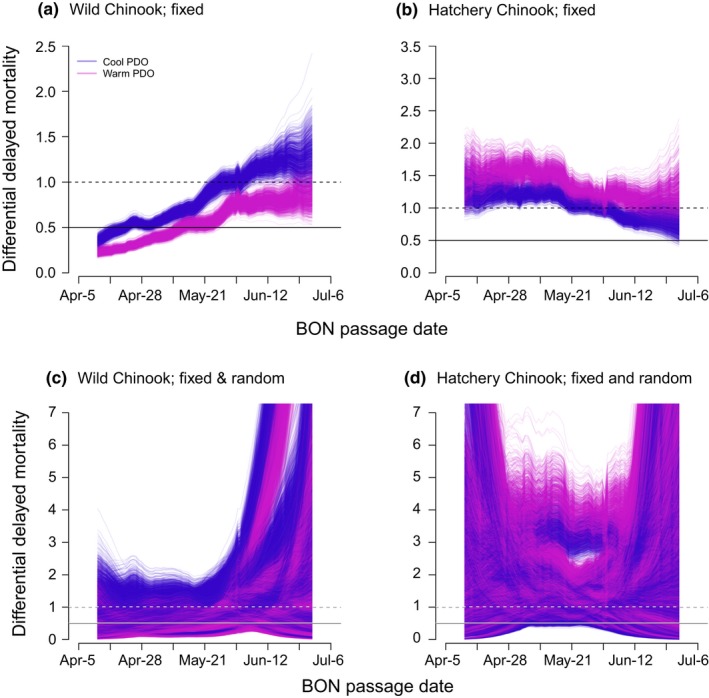
Differential delayed mortality (*D = S*_transport_ */S*_run‐of‐river_) across cool/warm PDO phases simulated from the model‐averaged GLMM of Chinook salmon survival (*S*) with (a and b) fixed effects parameters only, and (c and d) fixed effects and random effects parameters, for wild and hatchery rear‐types. Horizontal lines represent thresholds for which an advantage or disadvantage of transportation occurs in survival after the hydropower system (i.e., *D* = 1), or inclusive of the hydropower system (*D* ≈ 0.5)

The benefits and detriments of transportation represented by *D* were different for wild and hatchery Chinook salmon (Figure 5a,b). The value of transportation for wild Chinook went from detrimental to beneficial over the migration season. In contrast, transportation generally benefited hatchery Chinook across the season. The advantages of transportation were clearer when including the hydropower system survival and comparing *D* to a threshold of 0.5. Yet, detrimental effects from transportation to wild Chinook salmon were still apparent in April. When including the simulated random effects, a seasonal increase in *D* remained in wild fish (Figure 5c), whereas hatchery fish showed seasonally decreasing, increasing, and flat patterns (Figure 5d). The range of uncertainty in *D* from the simulations including random effects spanned across 1 and 0.5 (i.e., no clear benefit or detriment of transportation) throughout the season for both wild and hatchery rear‐types (Figure 5c,d). Thus, other influences on survival that were not explicitly tested in this study were captured by the random effects.

## 4 DISCUSSION

**Figure 6:**
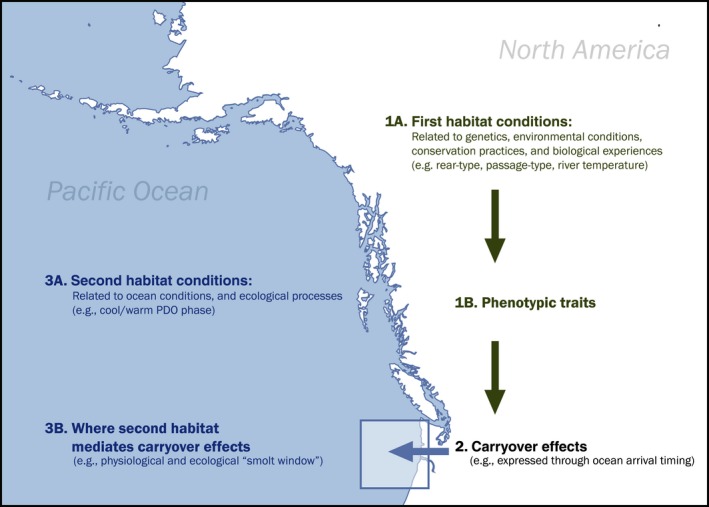
Conceptual diagram of freshwater carryover effects into the ocean with examples related to salmonids migrating through a hydropower system: (1a) First habitat experiences (1b) can be expressed as phenotypic traits (2) that can carry over into the next habitat. (3a) The strength of selection on the pool of traits will then depend on the conditions in the new habitat, (3b) particularly when juveniles leave the freshwater environment and enter the ocean

Conservation efforts across wide‐ranging taxa can be improved by considering ecological carryover effects across life stages (O'Connor & Cooke, 2015). For example, experiences during rearing, overwintering, and migration in the first habitat can result in changes in performance and survival in later life stages. The effects could involve physiological processes (Davy et al., 2016; McKinnon, Stanley, & Stutchbury, 2015; Midwood, Larsen, Boel, Aarestrup, & Cooke, 2015), genetic influences (Ceriani et al., 2015), and conservation practices (Holsman et al., 2012). Figure 6 demonstrates the complexity of interactions.

The concept of carryover effects is particularly powerful for the restoration of Columbia River salmonids. While many studies from the Columbia River Basin tested ocean conditions as a covariate to Chinook salmon productivity and abundances (Burke et al., 2013; Petrosky & Schaller, 2010), few have looked at interactions among freshwater and ocean covariates on survival linked to migration timing (Holsman et al., 2012). Our current study identified that the relative benefit of transportation on survival depends on the cool/warm PDO phase (Figure 5).

### 4.1 Transportation decisions

From a carryover effects perspective, we demonstrated that the effectiveness of transporting juvenile Chinook salmon on subsequent life stage survival differed among rear‐types. Transporting wild juveniles had a greater positive impact on their survival in cool than warm PDO years. Given general favorable conditions during cool years, earlier ocean arrival timing can result in greater opportunity to reach higher growth rates than that conferred in the river environment (Weitkamp et al., 2015). As well, conditions experienced during barge transportation may be more stressful to wild fish in warm than cool years. Effects can include disease (Dietrich et al., 2011) and stress from cotransportation with juvenile steelhead (Sandford, Zabel, Gilbreath, & Smith, 2012).

In contrast, transportation was more beneficial to hatchery Chinook salmon in warm than cool years. It is possible that transportation helped to minimize stressful exposure of hatchery juveniles to lower flow and warmer river conditions that generally occur in years with a positive (warm) PDO index (Mote, 2003; current study). This pattern supports the hypothesis that reducing stressful conditions experienced by juveniles in one life stage can result in higher survival in later life stages. The opposing pattern between rear‐types may occur because hatchery fish do not survive as well as wild fish in the early marine environment during years of poor ocean conditions (Beamish et al., 2012). Hatchery fish may need additional relief from stressful hydrosystem conditions that then carry over to benefit survival in subsequent life stages. Additionally, harvest is more intense on hatchery fish. Although we are not aware of differential harvest between transported and run‐of‐river fish, this may contribute to the rear‐type differences observed.

Differing patterns among rear‐types, populations, and species can make decisions about conservation strategies challenging. Generally, wild fish are the focus of conservation, and hatchery fish are produced to help enhance fishery production (Naish et al., 2007). Thus, the decision to transport in cool years could be more heavily weighted in favor of the positive effects on wild fish, but at some cost to the survival of hatchery fish. The effects of transportation on survival in hatchery Chinook salmon are generally positive or neutral (i.e., *D *≥* *1) across warm and cool PDO years. Thus, transporting hatchery Chinook salmon in cool years is not necessarily a counterproductive mitigation strategy for hatchery fish, but rather a lost opportunity to increase their posthydrosystem survival. Overall, the PDO index can serve as an annual baseline to help predict whether transportation will be a beneficial or disadvantageous conservation strategy.

### 4.2 Annual patterns

The basis for large‐scale effects of the PDO index stems from relationships of climate indices with oceanographic and ecosystem processes. Local ecosystem dynamics (e.g., high‐lipid copepods at lower trophic levels relate to higher salmon survival) are linked to large‐scale oceanographic forcings as indexed by the PDO (Bi et al., 2011). As well, salmon recruitment links to NPGO through food web processes and feeding ecology (Hertz et al., 2016). Multiple ecological pathways link large‐scale climate indices to salmon recruitment, but the PDO appears to be more influential than the NPGO or Oceanic Niño indices (Malick, Cox, Peterman, Wainwright, & Peterson, 2015). Our study extends the importance of the PDO index as a mediator of carryover effects. Although specific ocean conditions are difficult to forecast, our study shows that even a categorical climate index provides practical and actionable information for decision makers.

Forecasting ocean conditions undoubtedly provides information for decisions on salmonid conservation. For example, Chittenden et al. (2010) suggested that upwelling forecasts are useful for timing of hatchery releases to improve marine survival. However, the timeliness, accuracy, and certainty of forecasts are essential for effective conservation. Implementation of the information from the current study will require real‐time or forecasted fish, river, and ocean data. Also of value is forecasting a simple categorical index of a positive or negative PDO phase. This is possible given the high degree of autocorrelation within a 6‐month window and ongoing advances in oceanography (Di Lorenzo et al., 2013; Newman et al., 2016).

### 4.3 Seasonal patterns

In addition to the annual, large‐scale conditions of the ocean, there are within‐season effects. Anadromous fishes have evolved to enter the ocean at a time of favorable conditions (i.e., physiological and ecological “smolt window”; McCormick, Hansen, Quinn, & Saunders, 1998; Thorstad et al., 2012). Correspondingly, migration timing is a major determinant of survival for salmonids (Jonsson & Jonsson, 2014; McCormick et al., 1998; Scheuerell et al., 2009). In the river, migration timing can be related to temperature and flow (McCormick et al., 1998; Petrosky & Schaller, 2010; Scheuerell et al., 2009; Thorstad et al., 2012). More specifically, increased river temperatures can result in faster physical and physiological changes and earlier migration (Russell et al., 2012; Sykes, Johnson, & Shrimpton, 2009; Zydlewski, Haro, & McCormick, 2005). At ocean entrance, timing can be an index of both the environmental conditions and the predator and prey communities the juveniles encounter (Emmett, Krutzikowsky, & Bentley, 2006; Hvidsten et al., 2009; Logerwell et al., 2003; Wells et al., 2016). Although a detailed study of biological and ecological processes that affect juvenile‐to‐adult survival was beyond the scope of the study, we identified that migration timing continues to be a covariate of significant importance.

Further research on processes underlying migration timing and survival will arm decision makers with information to improve conservation strategies. Numerous studies revealed that freshwater–marine carryover effects on salmon survival generally involve physiological development (Drenner et al., 2012; Russell et al., 2012), fish size (Jonsson & Jonsson, 2014; Zabel & Achord, 2004), and growth‐ and size‐selective mortality (Miller et al., 2014; Woodson et al., 2013). Altogether, these and other studies emphasize the importance of juveniles entering the ocean in optimal physiological and ecological conditions (Hvidsten et al., 2009; McCormick et al., 1998; Wells et al., 2016). One common underlying factor related to these physiological and ecological processes is temperature. Increasing temperatures over the last several decades correspond to juveniles migrating earlier and at smaller sizes and younger ages, particularly at northern latitudes (Kovach, Joyce, Echave, Lindberg, & Tallmon, 2013; Otero et al., 2014; Russell et al., 2012). Thus, understanding mechanisms of migration timing will be especially important with river and ocean environments warming at different rates as climate changes (IPCC, 2014). Notably, correlations between river migration timing and ocean survival will likely change (Kennedy & Crozier, 2010).

The seasonal covariates in this study have been highlighted in other studies: higher survival with increased river flow, faster migration and development with increased temperature, and high predation in the estuary and plume areas (Brosnan et al., 2014; McCullough et al., 2009; Petrosky & Schaller, 2010). Yet, much uncertainty remains with the random effects of year and their interactions with migration timing. Other modeling approaches that capture more complex ecosystem dynamics may improve forecasting carryover effects. These include dynamic linear modeling (Scheuerell & Williams, 2005), nonlinear models with generalizable thresholds for management (Hunsicker et al., 2016), and models of intermediate complexity that incorporate data closely reflecting bottom‐up and top‐down processes (Wells et al., 2017).

In addition, the patterns of carryover effects captured in random effects can be further clarified by separating the adult upstream migration life stage from the ocean life stage. Increased rates of straying are known to occur for transported fishes (Bond et al., 2017; Keefer & Caudill, 2014). We used a detection site of adult returns past known locations of straying to account for this behavior. However, river conditions during upstream migration can stimulate straying (e.g., for thermal refuge). The lack of covariates during upstream migration could explain some of the patterns expressed as random effects in our study. There are thus direct effects of conditions experienced in each habitat and carryover effects from a previous habitat that are mediated by conditions in the current habitat.

### 4.4 Final thoughts

A growing concern in conservation is larger and more frequent mismatches between migration timing and timing of resources in subsequent habitats (Both, Bouwhuis, Lessells, & Visser, 2006; O'Connor et al., 2014). To address this concern effectively, our study showed that the effects of migration timing on survival need to be interpreted in context of carryover effects. Furthermore, migration timing can be an index of underlying processes involving the timing, quantity, and quality of resources, competitors, and predators across habitats. Thus, data on migration timing and the underlying processes can be particularly important information for deciding when and how to release juveniles in coastal fishery practices (e.g., restocking, stock enhancement, and sea ranching; Bartley & Bell, 2008). Explicitly considering carryover effects with pre‐ and within‐season data can help target conservation efforts more effectively. As shown in our study, considering large‐scale marine conditions helped to identify which years and when in the season it is more effective to transport juveniles. Applying large‐scale ocean forecasts with knowledge of stock‐ and passage‐specific carryover effects may provide a useful and practical strategy for buffering the effects of a changing climate on anadromous fishes.

## DATA ACCESSIBILITY

The datasets used in this manuscript are available at Columbia River Basin, School of Aquatic and Fishery Sciences, University of Washington (www.cbr.washington.edu).

## CONFLICT OF INTEREST

None declared.

## AUTHOR CONTRIBUTIONS

JG, JF, and RZ conceived the ideas and designed methodology. BS queried the PIT‐tagged fish data. AB modeled the estuary and plume data. JG queried the other environmental covariates and analyzed the data. JG, JA, and RZ led the writing of the manuscript. All authors contributed critically to the drafts and gave final approval for publication.
