# Supplementary material for: Conservation planning for freshwater–marine carryover effects on Chinook salmon survival
Source: Ecol Evol. 2017 Nov 28;8(1):319–32. doi: 10.1002/ece3.3663 (PMC5756849; doi:10.1002/ece3.3663)
Supplement: Supplementary file 1 [file ECE3-8-319-s001.docx]

**­­Conservation planning for freshwater-marine carryover effects on Chinook salmon survival**

Jennifer L. Gosselin, Richard W. Zabel, James J. Anderson, James R. Faulkner, António M. Baptista, and Benjamin P. Sandford

**Supporting Information**

Table S1. Correlation matrix of covariates. Blue highlights *r* < –0.5 and red highlights *r* > 0.5 to show highly correlated covariates (Dormann et al. 2013). Covariates beginning with “*z*” are normalized to mean 0 and standard deviation 1. Covariates beginning with “*r*” are residuals after accounting for a linear relationship with day-of-year of passage through Bonneville Dam (*z.d*; i.e., for river temperature [*r.t*] and sea surface temperature [*r.T*]), or accounting for a linear relationship with flow at Bonneville Dam (Flow [*F*]; i.e., for salt intrusion length (*r.E*) and plume volume [*r.V*]). For further description of covariates, see Table 1.

|  | ***z.d*** | ***z.t*** | ***r.t*** | ***z.T*** | ***r.T*** | ***f*** | ***z.E*** | ***r.E*** | ***z.V*** | ***r.V*** | ***z.U*** |
| --- | --- | --- | --- | --- | --- | --- | --- | --- | --- | --- | --- |
| ***z.d*** |  | 0.94 | 0.00 | 0.84 | 0.00 | 0.25 | -0.10 | 0.19 | 0.02 | -0.14 | 0.31 |
| ***z.t*** |  |  | 0.34 | 0.90 | 0.20 | 0.10 | 0.08 | 0.29 | -0.11 | -0.19 | 0.31 |
| ***r.t*** |  |  |  | 0.31 | 0.58 | -0.41 | 0.52 | 0.31 | -0.38 | -0.18 | 0.06 |
| ***z.T*** |  |  |  |  | 0.54 | 0.11 | 0.07 | 0.28 | -0.08 | -0.16 | 0.19 |
| ***r.T*** |  |  |  |  |  | -0.19 | 0.29 | 0.23 | -0.18 | -0.09 | -0.14 |
| ***f*** |  |  |  |  |  |  | -0.84 | 0.00 | 0.55 | 0.00 | 0.05 |
| ***z.E*** |  |  |  |  |  |  |  | 0.55 | -0.69 | -0.28 | -0.14 |
| ***r.E*** |  |  |  |  |  |  |  |  | -0.42 | -0.50 | -0.17 |
| **z.V** |  |  |  |  |  |  |  |  |  | 0.83 | 0.18 |
| ***r.V*** |  |  |  |  |  |  |  |  |  |  | 0.18 |
| ***z.U*** |  |  |  |  |  |  |  |  |  |  |  |

Figure S1. Number of passive-integrated-transponder-tagged Chinook salmon juveniles (or smolts) originating above Lower Granite Dam and passing Bonneville Dam. Panels show the different groups by rear-type (wild or hatchery) and passage-type (run-of-river [ROR] or transported) combinations.

Figure S2. Number of passive-integrated-transponder-tagged Chinook salmon adults originating above Lower Granite Dam. These fish were passing Bonneville Dam as juveniles and Lower Granite Dam as adults. Panels show the different groups by rear-type (wild or hatchery) and passage-type (run-of-river [ROR] or transported) combinations.

Figure S3. As Fig. 4, for wild, transported Chinook salmon.

Figure S4. As Fig. 4, for hatchery, run-of-river Chinook salmon.

Figure S5. As Fig. 4, for hatchery, transported Chinook salmon.

**Reference**

Dormann, C. F., Elith, J., Bacher, S., Buchmann, C., Carl, G., Carré, G., García Marquéz, J. R., Gruber, B., Lafourcade, B., Leitão, P. J., Münkemüller, T., McClean, C., Osborne, P. E., Reineking, B., Schröder, B., Skidmore, A. K., Zurell, D., and Lautenbach, S. 2013. Collinearity: a review of methods to deal with it and a simulation study evaluating their performance. Ecography 36:27-46. doi: 10.1111/j.1600-0587.2012.07348.x
